# Mediating role of resilience in the relationship between COVID-19 related stigma and mental health among COVID-19 survivors: a cross-sectional study

**DOI:** 10.1186/s40249-023-01074-3

**Published:** 2023-03-28

**Authors:** Weijun Xiao, Xiaoyang Liu, Hao Wang, Yiman Huang, Zhenwei Dai, Mingyu Si, Jiaqi Fu, Xu Chen, Mengmeng Jia, Zhiwei Leng, Dan Cui, Winnie W. S. Mak, Liming Dong, Xiaoyou Su

**Affiliations:** 1 School of Population Medicine and Public Health, Chinese Academy of Medical Sciences & Peking Union Medical College, 31 BeiJiGe San Tiao, Dongcheng District, Beijing, China; 2 Department of Pulmonary and Critical Care Medicine, National Center for Respiratory Medicine, Center of Respiratory Medicine, National Clinical Research Center for Respiratory Diseases, China-Japan Friendship Hospital, Beijing, China; 3 Department of Pulmonary and Critical Care Medicine, The 2nd Affiliated Hospital of Harbin Medical University, Harbin Medical University, Harbin, China; 4 Diversity and Well-Being Laboratory, Department of Psychology, The Chinese University of Hong Kong, Shatin, New Territories Hong Kong, China

**Keywords:** COVID-19, Survivor, Mental health, Resilience, Mediating affect, Stigma

## Abstract

**Background:**

The global coronavirus disease 2019 (COVID-19) has caused many negative effects on physical and mental health of patients who have survived COVID-19. Apart from some long-lasting physical sequelae, those COVID-19 survivors are also suffering stigma and discrimination at different levels around the world. The current study aims to assess the role resilience played in stigma and mental disorders among COVID-19 survivors.

**Methods:**

The cross-sectional study was carried out among former COVID-19 patients in Jianghan District (Wuhan, China) from June 10 to July 25, 2021. The demographic questions, the Impact of Events Scale-Revised, the Generalized Anxiety Disorder Questionnaire, the Patient Health Questionnaire, the Resilience Style Questionnaire and the Short Version of COVID-19 Stigma Scale of 12 items were used to collect relevant information of the participants. Descriptive analyses, Pearson correlation analysis and Structural Equation Modeling were used to make data description and analysis.

**Results:**

A total of 1541 out of 1601 COVID-19 survivors (887 females and 654 males) were included in the analysis. Perceived stigma of those COVID-19 survivors correlates significantly with anxiety (r = 0.335, *P* < 0.001), depression (r = 0.325, *P* < 0.001) and post-traumatic stress disorder (PTSD) (r = 0.384, *P* < 0.001). It has a direct effect on COVID-19 survivors’ anxiety (β = 0.326, *P* < 0.001), depression (β = 0.314, *P* < 0.001), PTSD (β = 0.385, *P* < 0.001) and their resilience (β = − 0.114, *P* < 0.01). Resilience partially mediated the association between perceived stigma and anxiety (β = 0.020, *P* < 0.01), depression (β = 0.020, *P* < 0.01), and PTSD (β = 0.014, *P* < 0.01) among COVID-19 survivors.

**Conclusion:**

Stigma has a significant negative impact on mental health, while resilience plays a mediator role in the relationship between stigma and mental health among COVID-19 survivors. Based on our study, we suggested that when designing psychological interventions for COVID-19 survivors, consideration should be taken into account to reduce stigma and improve resilience.

**Supplementary Information:**

The online version contains supplementary material available at 10.1186/s40249-023-01074-3.

## Background

The global coronavirus disease 2019 (COVID-19) was caused by a virus which has been named severe acute respiratory syndrome coronavirus 2 (SARS-CoV-2) [1]. The pandemic not only affected social activities due to quarantine and lock down regulation [2, 3], but also produced many negative effects on physical and mental health of people all over the world [4–6]. After virus infection, different types of damage would occur in many organs of the COVID-19 patients, especially in the brain [7]. As reported by previous studies, there have been many long-lasting sequelae of the COVID-19 survivors, including chronic fatigue, reduced physical capacity, muscles weakness, increased depression, anxiety, post-traumatic stress disorder (PTSD), and sleep problems [8–10]. A cross-sectional survey in Vietnam showed that the overall prevalence rates of PTSD, anxiety and depression among COVID-19 patients were 22.9%, 11.2% and 17.4%, respectively [11]. Therefore, the current study will pay more attention to the psychiatric sequelae and their influencing factors.

Patients who have survived COVID-19 are facing stigma and discrimination all over the world [12]. Stigma is a social process set to exclude those who are perceived to be a potential source of disease and may pose threat to the effective social activities and normal lives [13, 14], and it is an important factor related to mental distress during COVID-19 pandemic [15, 16]. A nationwide cross-sectional study which was carried out during the early stage of the pandemic in China showed that patients with COVID-19 and general residents in Wuhan suffered stigma both at individual and community levels [17]. What’s more, the COVID-19-related stigma will affect different segments of the society, including patients, their families and health care providers, which could disrupt the identification and surveillance of patients and consequently exert a negative influence on the control and management of COVID-19 pandemic [18]. A cross-sectional study in Wuhan, China shows that the experience of COVID-19-related discrimination is indirectly associated with anxiety, depression, and insomnia, in which shame and internalized stigma produced a complete mediating effect [19]. Another study found that stigma can influence mental health both directly and indirectly through the mediating effect of resilience of the physical disabled population in China [20].

Resilience refers to a person’s ability to withstand or adaptively recover from adversities, and it is closely related to psychological distress, such as depression, anxiety, stress, and posttraumatic stress [21]. Resilience is a key protective factor in mental health which would protects against negative psychological outcomes [22], and enhancing resilience in the face of the COVID-19 pandemic has important implications in terms of improving mental health conditions among psychiatric patients and general adults [23, 24]. Additionally, resilience has been identified as a mediator between stressful events and psychological well-being in previous studies. For instance, it has been proved an effective mediator between perceived stress and symptoms like anxiety, depression and psychological distress among nurses [25], as well as between pandemic fatigue and clinical nurses’ mental health, sleep quality and job contentment during the COVID-19 pandemic [26]. Another study has also found that resilience plays a mediating role between stigma and health status of people living with HIV, and resilience as a protective factor might buffer the effect of internalized HIV stigma on health status [27]. Some other studies also suggested that resilience significantly moderated the association between stigma and depressive symptoms among young men who have sex with men in China [28, 29], and between perceived stigma and quality of life among people with inflammatory bowel disease [30]. Therefore, we could assume that resilience might mediate the relationship between stigma and psychological well-being among COVID-19 survivors in China.

**Fig. 1:**
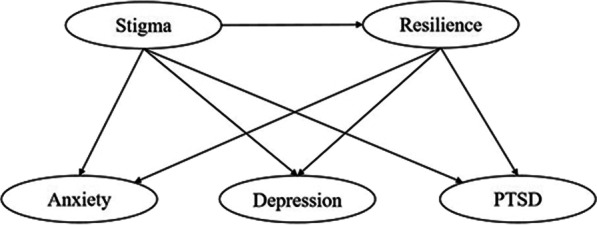
Conceptual framework of the potential mediating role of resilience between stigma and mental health among COVID-19 survivors

The current study is a cross-sectional survey designed to investigate the relationship between stigma and psychological well-being among COVID-19 survivors, and explored the possible mediating role of resilience at the same time. There were two primary study objectives. Firstly, the levels of stigma, resilience, and mental disorders (depression, anxiety and PTSD) and their possible associations were assessed among COVID-19 survivors. Secondly, the potential mediating role of resilience between stigma and psychological well-being were investigated among this population. The study hypotheses were structured and described as follows: COVID-19 survivors’ stigma is positively correlated with symptoms of anxiety, depression and PTSD; resilience is negatively correlated with stigma, anxiety, depression and PTSD; and finally, resilience acts as a mediator between stigma, symptoms of anxiety, depression and PTSD (Fig. 1).

## Methods

### Sampling and participants

The cross-sectional study was carried out among former COVID-19 patients in Jianghan District (Wuhan, China) from June 10 to July 25, 2021. According to the electronic medical records of the Health Bureau of Jianghan District and inclusion criteria, a total of 3059 COVID-19 patients were eligible for the study and they were infected with the original SARS-Cov-2 strain and were diagnosed between December 10, 2019 and April 20, 2020. Among them, 1601 COVID-19 survivors were invited for a questionnaire survey on their mental health status when they were receiving clinical re-examination, and 1541 completed the survey and were included in the analysis. All investigators and support staff in this study were trained based on the same protocol and were required to have an educational background in medicine or public health. Digital informed consent was obtained from all individuals to ensure their voluntary participation. Self-administered electronic questionnaires and digital consent were sent to patients through Redcap, an online survey platform (https://dce.cicams.ac.cn/redcap/). See details of the questionnaries in Additional file 2.

The participants have to meet the following inclusion criteria: (1) Have a history of SARS-CoV-2 infection. (2) Proficiency in Chinese. (3) Be able to independently cooperate with doctors to complete various scale assessments and the questionnaire. Those who meet any of the below exclusion criteria would be excluded: (1) Difficult to cooperate with the questionnaire survey. (2) Have extremely serious heart, brain, kidney, lung, liver, and other medical diseases. (3) Have a history of clinically diagnosed mental disorder or suicidal tendency before COVID-19 infection. (4) Women who are pregnant or breastfeeding.

### Demographics and measures

Demographic characteristics, including age, gender, education, marital status, and items on COVID-19 infection, such as clinical classification of those COVID-19 survivors was collected.

The Resilience Scale Questionnaire of 16 items was used to measure individual resilience. The items in this scale were scored on a 5-point Likert-type response format which was graded from ‘1’ (never) to ‘5’ (always) [31]. Higher total scores indicate greater ability to recover from negative events. In this study, the Cronbach’s alpha of the instrument was 0.975.

The Short Version of COVID-19 Stigma Scale of 12 items was used to measure the perceived stigma of COVID-19 survivors in previous 2 weeks. The scale’s items were scored on a 4-point Likert scale (0 = strongly disagree, 4 = strongly agree) [32, 33]. The total scores ranged between 0 and 48. Higher total scores indicate greater stigmatization. The Stigma Scale contains four dimensions measuring personalized stigma, disclosure concerns, concerns about public attitude and negative self-image. In this study, the Cronbach’s alpha of the instrument was 0.936.

The Impact of Events Scale-Revised (IES-R) of 22 items was used to screen posttraumatic stress symptoms in adults. The items of this instrument are rated on a 5-point Likert scaled from 0 to 4 [34, 35]. The IES-R contains three dimensions measuring intrusion, avoidance and hyperarousal. Respondents rate their degree of distress during the past 7 days after they have identified a specific stressful life event occurred to them. A total score of equal or above 35 can be regarded as positive PTSD symptoms. This instrument has been proved valid and reliable among COVID-19 patients [34]. In this study, the Cronbach’s alpha of the instrument was 0.965.

The Patient Health Questionnaire (PHQ-9) is a 9-item questionnaire which is used for screening depressive symptoms over the past 2 weeks [36]. The items of the PHQ-9 are rated on a 4-point Likert scaled ranging from 0 to 3. The total score is utilized to assess the degree of depression of participants, with scores of ≥ 5 indicating depression: Scores of 5–9 mild depression; 10–14 moderate depression; 15–19 moderately severe depression; and scores of ≥ 20 severe depression. In this study, the Cronbach’s alpha of the instrument was 0.914.

The Generalized Anxiety Disorder Questionnaire (GAD-7) consists of 7 items which are rated on a 4-point Likert scaled from 0 to 3. It was developed to measure the severity of generalized anxiety symptoms over the past 2 weeks [37]. The scores of the instrument range from 0 to 21. A cutoff score of ≥ 5 is recommended for considering significant anxiety symptoms, and scores between 5 and 9, 10 and 14, 15 and higher represent mild, moderate and severe anxiety symptoms respectively. In this study, the Cronbach’s alpha of the instrument was 0.951.

### Statistical analysis

Statistical analysis was performed in R v4.2.0 (R Core Team, Vienna, Austria). *P*-value of less than 0.05 was considered statistically significant (2-tailed tests). Descriptive analyses were performed to examine the participants’ demographic, clinical characteristics and the prevalence of psychological problems, which were described by frequencies (%) or means (SD), depending on the distribution of each variable. Pearson correlation coefficient was used to evaluate the association between the scores. Influencing factors of stigma among COVID-19 survivors were analyzed by linear regression, and significant variables (*P* ≤ 0.05) in the univariate linear regression analysis were further entered into the multiple linear regression analysis. After adjusting for age, body mass index (BMI), gender, education level, marital status, income, clinical classification, dwelling state, alcohol use and tobacco use, Structural Equation Modeling (SEM) using full information maximum likelihood estimators was applied to examine the mediation model, and the SEM was performed with bias-corrected confidence intervals (*CI*s) using 10,000 bootstrapped samples. Mediation analyses were conducted with Lavaan package in R.

We analyzed the association between stigma and symptoms of anxiety, depression and PTSD among COVID-19 survivors, and whether this was mediated by resilience. The fitness between the current data and the hypothesized model was assessed through the following indicators: (a) the root-mean-square error of approximation (RMSEA); (b) the standardized root-mean square residual (SRMR); (c) the comparative fit index (CFI), and (d) the Tucker–Lewis index (TLI). The RMSEA and the SRMR with values below 0.08, and CFI and TLI with values over 0.9, indicate a good fit.

## Results

### Participants’ characteristics and prevalence of psychological symptoms

A total of 1541 COVID-19 survivors were included in the analysis. The study participants consisted of 654 males (42.4%) and 887 females (57.6%). The mean age was 57.5 ± 12.4 years and BMI was 24.5 ± 3.4. Most of the participants (85.2%) were married, 61.5% of them had annual family income less than CNY 60,000 in 2020, 3.2% used to be treated at intensive care unit (ICU) and 12.7% of the participants were living alone. In this study, 234 (15.2%) were positively indicated for PTSD, 418 (27.1%) and 558 (36.2%) reported mild to severe levels of anxiety and depression symptoms (Table 1).

**Table 1:** Baseline characteristics of the study participants (*n* = 1541)

Variables	Mean (*SD*) or *n* (%)
Age (mean, *SD*)	57.5 (12.4)
BMI (mean, *SD*)	24.5 (3.4)
Gender	
Male	654 (42.4%)
Female	887 (57.6%)
Marital status	
Married	1313 (85.2%)
Unmarried/divorced/widowed	228 (14.8%)
Annual household income for 2020 (CNY)	
< 60,000	947 (61.5%)
≥ 60,000	594 (38.5%)
Dwelling state	
Living alone	195 (12.7%)
Living together	1346 (87.3%)
Education level	
Senior high school or below	1082 (70.2%)
Above senior high school	459 (28.8%)
Underlying diseases	
Yes	896 (58.1%)
No	645 (41.9%)
Experience at intensive care unit	
Yes	49 (3.2%)
No	1492 (96.8%)
Clinical classification of COVID-19 patients	
Asymptomatic	90 (5.8%)
Mild	1113 (72.2%)
Moderate	151 (9.8%)
Critically severe	187 (12.2%)
Tobacco use	
Yes	193 (12.5%)
No	1348 (87.5%)
Frequency of alcohol use per week	
< 2	1396 (90.6%)
≥ 2	145 (9.4%)
Post-traumatic stress disorder	
Yes	234 (15.2%)
No	1307 (84.8%)
Anxiety	
No	1123 (72.9%)
Mild	331 (21.5%)
Moderate	52 (3.4%)
Severe	35 (2.2%)
Depression	
No	983 (63.8%)
Mild	357 (23.2%)
Moderate	124 (8.0%)
Moderate severe	56 (3.6%)
Severe	21 (1.4%)

Data are presented as *n* (%) or mean (SD)

### Descriptive statistics and Pearson correlations of COVID-19 related stigma, resilience and psychological symptoms

Descriptive statistics and correlations between COVID-19 related stigma, resilience, anxiety, depression and PTSD were presented. The mean COVID-19 related stigma was 27.8 ± 7.3 (scores) and the mean resilience was 57.0 ± 14.0 (scores) (Table 2). According to the Pearson correlations, the participants’ perceived stigma correlated negatively with resilience and positively with PTSD, anxiety and depression. COVID-19 survivors’ perceived stigma correlated significantly with anxiety (r = 0.335, *P* < 0.001), depression (r = 0.325, *P* < 0.001) and PTSD (r = 0.384, *P* < 0.001). The participants who perceived a high level of stigma also indicated more symptoms of anxiety, depression and PTSD. Resilience correlated negatively with perceived stigma (r = − 0.129, *P* < 0.001), anxiety (r = − 0.207, *P* < 0.001), depression (r = − 0.213, *P* < 0.001) and PTSD (r = − 0.152, *P* < 0.001) (Table 2).

**Table 2:** Descriptive statistics and Pearson correlations of COVID-19 related stigma, resilience and psychological symptoms

Variables	M	*SD*	Min	Max	Stigma	Resilience	Anxiety	Depression	PTSD
Stigma	27.8	7.3	12	48	1.000				
Resilience	57.0	14.0	16	80	− 0.129***	1.000			
Anxiety	2.9	4.0	0	21	0.335***	− 0.207***	1.000		
Depression	4.2	4.9	0	27	0.325***	− 0.213***	0.824***	1.000	
PTSD	16.7	16.9	0	88	0.384***	− 0.152***	0.686***	0.729***	1.000

*M* mean, *Min* minimum, *Max* maximum, *SD* standard deviation, *PTSD* post-traumatic stress disorder

*Significant correlation, *P* value < 0.05

**Significant correlation, *P* value < 0.01

***Significant correlation, *P* value < 0.001

### Factors associated with stigma

According to multiple linear regression, there were five factors associated with stigma among COVID-19 survivors, including age (β = 0.112, *P* < 0.001), gender (β = 1.473, *P* < 0.001), income (β = − 0.821, *P* = 0.038), education level (β = − 2.113, *P* < 0.001) and severity of COVID-19 (β = 0.520, *P* = 0.028). More details were showed in Additional file 1: Appendices S1 and S2).

### Mediating role of resilience between stigma and psychological symptoms

**Fig. 2:**
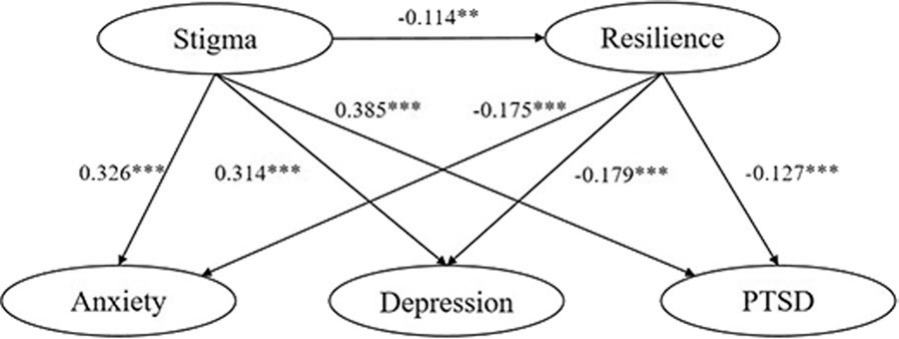
Structural equation model for the mediating role of resilience between stigma and mental health among COVID-19 survivors with standardized estimates. *Significant at level *P* < 0.05, **Significant at level *P* < 0.01, ***Significant at level *P* < 0.001. *PTSD* post-traumatic stress disorder

The SEM was used to test the hypothesized model. The standardized fit indices indicated that the model was appropriate: the RMSEA was 0.060, the SRMR was 0.054, the CFI was 0.993, and the TLI was 0.988. The standardized estimates for the structural model are shown in Fig. 2.

The relationships between the variables were examined. Mediation analysis was conducted to test the hypothesized correlations between the perceived stigma and COVID-19 survivors’ resilience, anxiety, depression and PTSD. The results indicated that perceive stigma has a direct effect on COVID-19 survivors’ anxiety (β = 0.326, *P* < 0.001), depression (β = 0.314, *P* < 0.001) and PTSD (β = 0.385, *P* < 0.001). Furthermore, the results also identified that perceived stigma of COVID-19 survivors had a significant direct effect on their resilience (β = − 0.114, *P* < 0.01). The statistical analyses showed that resilience partially mediated the association between perceived stigma and COVID-19 survivors’ anxiety (β = 0.020, *P* < 0.01), depression (β = 0.020, *P* < 0.01), and PTSD (β = 0.014, *P* < 0.01) (Tables 3 and 4; Fig. 2).

**Table 3:** Parameters estimated for the model of the mediating role of resilience between stigma and mental health among COVID-19 survivors

Variables	B	Std. Error	β	*t*-value	*P* (> |t|)
Stigma → resilience	− 0.533	0.164	− 0.114	− 3.256	0.001
Stigma → depression	1.007	0.101	0.314	9.983	< 0.001
Resilience → depression	− 0.122	0.017	− 0.179	− 7.259	< 0.001
Stigma → anxiety	0.847	0.080	0.326	10.587	< 0.001
Resilience → anxiety	− 0.097	0.013	− 0.175	− 7.297	< 0.001
Stigma → PTSD	1.448	0.126	0.385	11.535	< 0.001
Resilience → PTSD	− 0.102	0.018	− 0.127	− 5.535	< 0.001

*B* unstandardized estimate, *β* standardized estimate, *PTSD* post-traumatic stress disorder

**Table 4:** Direct, indirect and total effect for mediation models

Variables	B	β	95% *CI*	*P*-value
ACME^a^	0.065	0.020	(0.029, 0.118)	0.003
ADE^a^	1.007	0.314	(0.814, 1.213)	< 0.001
Total effect^a^	1.072	0.334	(0.880, 1.280)	< 0.001
ACME^b^	0.052	0.020	(0.023, 0.093)	0.003
ADE^b^	0.847	0.326	(0.699, 1.017)	< 0.001
Total effect^b^	0.899	0.346	(0.744, 1.069)	< 0.001
ACME^c^	0.054	0.014	(0.024, 0.102)	0.004
ADE^c^	1.448	0.385	(1.208, 1.698)	< 0.001
Total effect^c^	1.503	0.400	(1.258, 1.759)	< 0.001

Bootstrap *CI* estimation

*B* unstandardized estimate, *β* standardized estimate, *ACME* average causal mediation effects (indirect effect), *ADE* average direct effects, *CI* confidence Interval, *PTSD* post-traumatic stress disorder

^a^Depression as dependent variable

^b^Anxiety as dependent variable

^c^PTSD as dependent variable

## Discussion

The current study indicated that there were substantial psychosocial problems among COVID-19 survivors. The prevalence rates of anxiety, depression and PTSD among COVID-19 survivors were 27.1%, 36.2% and 15.2%, respectively. These rates were significantly higher than the rates noted in studies of the general adult population in China before COVID-19 pandemic (12-month generalized anxiety prevalence rates: 5.0%, depression prevalence rates: 3.6%, PTSD prevalence rates: 0.2%) [38], and the prevalence of anxiety and depression were lower than the results of two surveys conducted among COVID-19 patients in January and April 2020 in Wuhan, China [39, 40]. In addition, we could notice that the prevalence of depression (36.2%) was the highest among these psychological problems the participants experienced, and the high frequency of depressive symptoms associated with post-COVID-19 syndrome were also demonstrated by previous studies [41, 42]. The high prevalence rate of these psychological sequelae in COVID-19 survivors further highlighted the importance and urgency of addressing mental health of this group of people. Despite psychiatric sequelae in COVID-19 survivors were generally improved over time [43], the current study showed that their mental health status was still worse than those who had never been infected [44–46]. Therefore, the COVID-19 survivors need more psychological intervention or services. There were many intervention measures or methods to reduce psychological distress caused by COVID-19. For instance, mindfulness intervention aims to foster greater attention and awareness of present moment experience, and has been proved can improve mental health outcomes among various populations [47]. It has been confirmed that Internet-based mindfulness intervention can significantly improve the anxiety and depression symptoms of COVID-19 patients [48]. A randomized controlled trial also suggested that computerized cognitive behavioral therapy was an effective nonpharmacological treatment for symptoms of anxiety, depression, and insomnia among COVID-19 patients [49]. Moreover, online psychological intervention programs can be performed wherever the Internet is available, which may provide unique benefits in a socially-distanced world transformed by COVID-19.

With regard to the first hypothesis concerning the relationship between stigma and mental health, the current study found that COVID-19 survivors who perceived higher level of stigma had higher prevalence of anxiety, depression and PTSD. These results were consistent with previous studies, which showed that stigma was a risk factor for psychological problems among COVID-19 survivors. Feeling discriminated was a problem that could not be ignored among COVID-19 patients, and it would affect their mood [42]. A previous study conducted in China showed that stigma was one of the main concerns expressed by COVID-19 patients [50]. A review demonstrated that stigma was a risk factor for mental health problem among general population, patients with COVID-19 and health care providers during COVID-19, including anxiety, depression and stress [51]. A cross-sectional study among people who had recovered from COVID-19 in Iran suggested that mental health, COVID-19-related self-stigma, and mental quality of life were associated [52]. COVID-19-related stigma is commonly experienced among COVID-19 survivors even though the outbreak has been well-contained in China [53]. Moreover, the current study found that female survivors as well as those with older ages, severe COVID-19 symptoms, lower education and income levels were more likely to experience stigma [53–55]. Recently, the association between stigma and the duration of COVID-19 syndrome (long COVID) has been gradually recognized. And it is crucial to understand the recovery and disease progression journeys of COVID-19 survivors and subsequently to figure out how stigma shape long COVID [56]. Therefore, more researches should be conducted on this issue. Based on the knowledge and researches carried out, it is recognized that reducing stigma in COVID-19 survivors is very important to the mental health of them. There were some well-established programs to alleviate the health-related stigma, such as mass media campaigns and interventions [57, 58], family psychoeducation interventions [59], hallucination simulation interventions [60], education text interventions for target groups [61]. In addition, it has been suggested that the availability of data on health-related stigma and discrimination is critical for implementing interventions and programs to address them [62]. From another prospective, stigma is a social process engrained both at the individual and community levels, reducing the stigma of COVID-19 survivors requires the joint efforts of the whole society. Studies conducted in different populations have suggested several constructive methods, such as establishing and sticking to the social consensus theory [63], facilitating collaboration between the general and the stigmatized population [64] to enhance their positive interaction with each other [65], accelerating community rehabilitation programme development, and finally taking all possible measures to help the stigmatized group to reintegrate into the society [66].

Another finding in this study indicated that resilience had a significant impact on mental health among COVID-19 survivors. Resilience was negatively correlated with stigma, anxiety, depression and PTSD. This result is consistent with previously existing studies. Individuals with a high level of resilience have some dispositional qualities, such as tenacity, self-improvement and optimism [67], and resilience has been recognized as a significant protective factor against various mental and psychological stressors during disease outbreaks [68]. A study conducted in Spain suggested that resilience is negatively associated with depression during COVID-19 pandemic, and enhancing resilience in the face of the COVID-19 pandemic might have important implications in terms of improving mental health outcomes among psychiatric patients [23]. It has been proved that higher levels of resilience were associated with better mental health in elder population [69], and treatment interventions focused on enhancing psychological resilience can effectively treat PTSD [70]. There are many projects to enhance COVID-19 survivors’ resilience. For instance, interventions based on the concepts of positive psychology, supportive-expressive group therapy, behavioral therapy, or mindfulness can effectively promote psychological resilience [71]. Aerobic exercise can build resilience, thereby reducing the anxiety response [72]. In this study, the importance of resilience in previous knowledge was extended among COVID-19 survivors. Therefore, to understand and promote the resilience of COVID-19 survivors is important to maintain their psychological well-being, providing the long-COVID sequalae might still present among those COVID-19 survivors and the sporadic epidemic has yet to be curbed in a short period of time [73].

Through the mediating analysis, our third hypothesis that resilience plays a mediator role between perceived stigma and symptoms of anxiety, depression and PTSD was also demonstrated. In other words, COVID-19 survivors who had low scores on the perceive stigma might have high levels of resilience, which in turn would lead to better mental health. To the best of our knowledge, there is a lack of research on the mediating effect of resilience between stigma and mental disorders of COVID-19 survivors. A study conducted among college students indicated the partial mediating effect of resilience in the relationship between physical literacy and mental health [74]. The moderating role of resilience in the personality-mental health relationship during the COVID-19 pandemic also had been demonstrated [75]. In addition, there was evidence that resilience is a mediator between cardiorespiratory fitness and mental health-related quality of life [76]. The result of current study is consistent with previous studies and it has been proved that resilience can directly or indirectly alleviate the negative effect of stigma on COVID-19 survivors’ mental well-being and it seems to play a protective role in the relationship between stigma and depression, anxiety and PTSD. This result is helpful to better understand the mental health status of COVID-19 survivors and provide reference and guidance for their mental health intervention.

There were several limitations of this study. Firstly, it was a cross-sectional study and we can’t make statements regarding causality. Therefore, a longitudinal study should be carried out to verify the conclusions in the future. Secondly, this study was conducted more than 1 year after discharge of these COVID-19 patients, which may lead to recall bias, and at the same time, the self-reported questionnaires we used to assess COVID-19 related stigma, resilience and mental health were subject to the risk of response bias. Thirdly, PTSD, anxiety and depressive symptoms were measured by IES-R, GAD-7 and PHQ-9 using self-administered questionnaire instead of clinical diagnosis, which may influence the estimates. Fourthly, the participants of this study were recruited from one district of Wuhan city, which as a result, was not fully representative of all COVID-19 survivors in China. Fortunately, key demographic measures were assessed in this study and some confounding effects in demographics were statistically controlled within statistical analyses.

## Conclusion

Stigma is negatively related to psychological well-being of COVID-19 survivors even after discharged from hospitals for a long time, and resilience plays a mediator role in the relationship between stigma and those survivors’ psychological well-being. These findings deepened our understanding of the relationship and mediating mechanism among stigma, resilience and psychological well-being. When designing psychological interventions for COVID-19 survivors, consideration should be taken into account to reduce stigma and improve resilience.

## Supplementary Information

**Additional file 1: Appendix S1.** Univariate statistical analysis of influencing factors of COVID-19 related stigma among COVID-19 survivors by linear regression. **Appendix S2.** Multivariate statistical analysis of influencing factors of COVID-19 related stigma among COVID-19 survivors by multiple linear regression.

**Additional file 2:** Questionnaire on mental health status of patients recovered from COVID-19.

## Data Availability

The datasets generated and analyzed during the current study are not publicly available due to restriction, but the data are available from the corresponding author on reasonable request and with the permission of the institution.

## Abbreviations

COVID-19: Coronavirus disease 2019
ICU: Intensive care unit
HIV: Human immunodeficiency virus
IES-R: Impact of Events Scale-Revised
GAD-7: Generalized Anxiety Disorder Questionnaire-7
PHQ-9: Patient Health Questionnaire-9
PTSD: Post-traumatic stress disorder
M: Mean
Min: Minimum
Max: Maximum
*SD*: Standard deviation
CNY: Chinese Yuan
BMI: Body mass index
*CI*: Confidence interval
SEM: Structural Equation Modeling
ACME: Average causal mediation effects (indirect effect)
ADE: Average direct effects
RMSEA: Root-mean-square error of approximation
SRMR: Standardized root-mean square residual
CFI: Comparative fit index
TLI: Tucker–Lewis index
