# Supplementary material for: Mediating role of resilience in the relationship between COVID-19 related stigma and mental health among COVID-19 survivors: a cross-sectional study
Source: Infect Dis Poverty. 2023 Mar 28;12:27. doi: 10.1186/s40249-023-01074-3 (PMC10043530; doi:10.1186/s40249-023-01074-3)
Supplement: Supplementary file 1 — Additional file 1: Appendix S1. Univariate statistical analysis of influencing factors of COVID-19 related stigma among COVID-19 survivors by linear regression. Appendix S2. Multivariate statistical analysis of influencing factors of COVID-19 related stigma among COVID-19 survivors by multiple linear regression. [file 40249_2023_1074_MOESM1_ESM.docx]

Additional file 1

Appendix S1. Univariate statistical analysis of influencing factors of COVID-19 related stigma among COVID-19 survivors by linear regression.

| Variables | β (95% *CI*) | *P*-value |
| --- | --- | --- |
| **Age** | 0.146 (0.118, 0.175) | < 0.001*** |
| **BMI** | -0.097 (-0.204, 0.009) | 0.073 |
| **Gender** |  |  |
| Male | Reference |  |
| Female | 1.835 (1.104, 2.565) | < 0.001*** |
| **Marital status** |  |  |
| Married | Reference |  |
| Unmarried/divorced /widowed | 0.560(-0.465,1.584) | 0.284 |
| **Annual household income for 2020 (CNY)** |  |  |
| < 60,000 | Reference |  |
| ≥ 60,000 | -2.161 (-2.901, -1.421) | < 0.001*** |
| **Dwelling state** |  |  |
| Living alone | Reference |  |
| Living together | -0.200 (-1.294, 0.895) | 0.720 |
| **Education level** |  |  |
| Senior high school or below | Reference |  |
| Above senior high school | -3.626 (-4.401, -2.852) | < 0.001*** |
| **Underlying diseases** |  |  |
| Yes | Reference |  |
| No | -1.170 (-1.905, -0.434) | 0.002** |
| **Experience at ICU** |  |  |
| Yes | Reference |  |
| No | -1.598 (-3.67, 0.474) | 0.131 |
| **Clinical classification of COVID-19 patients** |  |  |
| Asymptomatic | 0.580 (0.095, 1.064) | 0.019* |
| Mild |  |  |
| Moderate |  |  |
| Critically severe |  |  |
| **Tobacco use** |  |  |
| Yes | Reference |  |
| No | 0.873 (-0.225, 1.972) | 0.119 |
| **Frequency of alcohol use per week** |  |  |
| < 2 | Reference |  |
| ≥ 2 | -0.586 (-1.832, 0.66) | 0.356 |

Note. * Significant at level *P* < 0.05, ** Significant at level *P* < 0.01, *** Significant at level *P* < 0.001. β: coefficient.

Appendix S2. Multivariate statistical analysis of influencing factors of COVID-19 related stigma among COVID-19 survivors by multiple linear regression.

| Variables | β (95% *CI*) | *P*-value |
| --- | --- | --- |
| **Age** | 0.112 (0.081, 0.144) | < 0.001*** |
| **Gender** |  |  |
| Male | Reference |  |
| Female | 1.473 (0.76, 2.185) | < 0.001*** |
| **Annual household income for 2020 (CNY)** |  |  |
| < 60,000 | Reference |  |
| ≥ 60,000 | -0.821 (-1.596, -0.047) | 0.038* |
| **Education level** |  |  |
| Senior high school or below | Reference |  |
| Above senior high school | -2.113 (-2.984, -1.241) | < 0.001*** |
| **Underlying diseases** |  |  |
| Yes | Reference |  |
| No | 0.150 (-0.608, 0.908) | 0.698 |
| **Clinical classification of COVID-19 patients** |  |  |
| Asymptomatic | 0.520 (0.055, 0.985) | 0.028* |
| Mild |  |  |
| Moderate |  |  |
| Critically severe |  |  |

Note. R-Squared (R^2^) = 0.100, Adjusted R-Squared (AR^2^) = 0.097, *P* < 0.001. * Significant at level *P* < 0.05, ** Significant at level *P* < 0.01, *** Significant at level *P* < 0.001. β: coefficient.
