# Supplementary material for: Mediating role of resilience in the relationship between COVID-19 related stigma and mental health among COVID-19 survivors: a cross-sectional study
Source: Infect Dis Poverty. 2023 Mar 28;12:27. doi: 10.1186/s40249-023-01074-3 (PMC10043530; doi:10.1186/s40249-023-01074-3)
Supplement: Supplementary file 2 — Additional file 2: Questionnaire on mental health status of patients recovered from COVID-19. [file 40249_2023_1074_MOESM2_ESM.docx]

Additional file 2: Questionnaire on mental health status of patients recovered from COVID-19.

**新冠肺炎康复患者心理健康状况调查**

您好！这是一项关于新冠肺炎康复患者的心理健康状况的调查，如果您同意参加本次调查，请勾选“我同意”选项；如果您不同意，请勾选“不同意”选项，且您的利益不会受到任何影响：

○我同意 ○不同意（无效问卷，跳转至结束作答）

**一、基本信息**

1. 您的年龄：_____（岁）
2. 您的性别：○男 ○女
3. 您的身高是：_______ (cm)；您的体重是_______ (kg)
4. 您的婚姻状况：○已婚 ○未婚/离婚/丧偶
5. 您的文化程度：

○高中及以下（包括中专/技校/职高） ○大学专科及以上

1. 2020年家庭年收入是？

○小于6万 ○ 6万及以上

1. 您目前和谁居住在一起？

○单独 ○父母/配偶/子女 ○其他（请说明）________

1. 您是否患有基础疾病？

○是 ○否

1. 您有吸烟的习惯吗？

○有 ○无

1. 您平时多久喝一次酒？

○每周次小于2次 ○每周2次及以上

1. 您感染新冠肺炎的日期：____年____ 月
2. 疫情出现的时候，您被确诊为以下哪种类型新冠肺炎？

○轻度 ○中度 ○重度 ○危重

1. 您是否进入过ICU（重症监护病房）进行治疗？

○是 ○否

**二、心理健康相关评估**

1.下面是人们在经历过有压力的生活事件刺激后所体验到的一些困扰，请您仔细阅读每个题目，并按自己过去7天的经验选择最能够形容每一种困扰对您影响的程度，在最符合您真实情况的选项处打“√”。以下提到的那件事是指：感染新冠肺炎有关经历。*-- 中文版的事件影响量表-修订版（Chinese version of Impact of Events Scale-Revised, IES-R）*

| 序号 | 条目 | 完全没有 | 很少出现 | 有时出现 | 常常出现 | 总是出现 |
| --- | --- | --- | --- | --- | --- | --- |
| 1 | 任何与那件事相关的事物都会引发当时的感受。 |  |  |  |  |  |
| 2 | 我很难安稳的一觉睡到天亮。 |  |  |  |  |  |
| 3 | 别的东西也会让我想起那件事。 |  |  |  |  |  |
| 4 | 我感觉我易刺激、易发怒。 |  |  |  |  |  |
| 5 | 每当想起那件事或其他事情使我记起它的时候，我会尽量避免使自己心烦意乱。 |  |  |  |  |  |
| 6 | 即使我不愿意去想那件事，也会想起它。 |  |  |  |  |  |
| 7 | 我感觉，那件事好像不是真的，或者从未发生过。 |  |  |  |  |  |
| 8 | 我设法远离一切能使我记起那件事的事物。 |  |  |  |  |  |
| 9 | 有关那件事的画面会在我的脑海中突然出现。 |  |  |  |  |  |
| 10 | 我感觉自己神经过敏，易受惊吓。 |  |  |  |  |  |
| 11 | 我努力不去想那件事。 |  |  |  |  |  |
| 12 | 我觉察到我对那件事仍有很多感受，但我没有去处理它们。 |  |  |  |  |  |
| 13 | 我对那件事的感觉有点麻木。 |  |  |  |  |  |
| 14 | 我发现我的行为和感觉，好像又回到了那个事件发生的时候那样。 |  |  |  |  |  |
| 15 | 我难以入睡。 |  |  |  |  |  |
| 16 | 我因那件事而有强烈的情感波动。 |  |  |  |  |  |
| 17 | 我想要忘掉那件事。 |  |  |  |  |  |
| 18 | 我感觉自己难以集中注意力。 |  |  |  |  |  |
| 19 | 令我想起那件事的事物会引起我身体上的反应。如：出汗、呼吸困难、眩晕和心跳。 |  |  |  |  |  |
| 20 | 我曾经梦到过那件事。 |  |  |  |  |  |
| 21 | 我感觉自己很警觉或很戒备。 |  |  |  |  |  |
| 22 | 我尽量不提那件事。 |  |  |  |  |  |

2.在过去两周，你有多大程度上受到以下问题困扰？请在最符合你真实情况的选项处打“√”。*-- 病人健康问卷（*[*Patient Health Questionnaire*](https://kns-cnki-net-443.webvpn.cams.cn/kns/detail/detail.aspx?QueryID=2&CurRec=5&recid=&FileName=SJPD2A1CFF0444D12A1519889829C4562897&DbName=SJPDLAST&DbCode=SJPD&yx=&pr=&URLID=&bsm=)*, PHQ-9）*

| 序号 | 条目 | 完全没有 | 有  几天 | 一半以上的天数 | 几乎每天 |
| --- | --- | --- | --- | --- | --- |
| 1 | 做任何事都觉得沉闷或者提不起劲或没有兴趣 |  |  |  |  |
| 2 | 情绪低落、沮丧或绝望 |  |  |  |  |
| 3 | 入睡困难、半夜会醒睡不安或睡得过多 |  |  |  |  |
| 4 | 感觉疲倦或活力不足 |  |  |  |  |
| 5 | 食欲不振或吃太多 |  |  |  |  |
| 6 | 不喜欢自己——觉得自己很糟、对自己失望或有负于家人的期望 |  |  |  |  |
| 7 | 难以集中精神做事，例如看报纸或看电视时 |  |  |  |  |
| 8 | 其他人反映你行动或说话缓慢；或者相反的，你比平时活动更多，烦躁、坐立不安或动来动去 |  |  |  |  |
| 9 | 有不如死掉或用某种方式伤害自己的念头 |  |  |  |  |

2.1如果您出现以上任何问题，这些问题对你的工作、日常处理家庭事务或与人相处来说有多少困难？

完全没有困难 B. 有一些困难 C. 非常困难 D. 极度困难

3.在过去两周，你有多经常受到以下问题困难？请在最符合你真实情况的选项处打“√”。*-- 广泛性焦虑障碍量表（Generalized Anxiety Disorder Questionnaire，GAD-7）*

| 序号 | 条目 | 完全没有 | 有  几天 | 一半以上的天数 | 几乎每天 |
| --- | --- | --- | --- | --- | --- |
| 1 | 感到紧张、不安或烦躁。 |  |  |  |  |
| 2 | 无法停止或控制担忧。 |  |  |  |  |
| 3 | 过分担忧不同的事情。 |  |  |  |  |
| 4 | 难以放松。 |  |  |  |  |
| 5 | 心绪不宁以至坐立不安。 |  |  |  |  |
| 6 | 容易心烦或易怒。 |  |  |  |  |
| 7 | 感到害怕，就像要发生可怕的事情。 |  |  |  |  |

4.以下的句子是对你行为及心态上的描述，请选择合适的答案。*-- 心理弹性量表（Resilience style questionnaire）*

| 序号 | 条目 | 从不 | 很少 | 有时 | 常常 | 总是 |
| --- | --- | --- | --- | --- | --- | --- |
| 1 | 我会挑战每个困难。 |  |  |  |  |  |
| 2 | 我相信在生活的谷底中我会很快反弹起来。 |  |  |  |  |  |
| 3 | 对于有价值的事，我会持之以恒。 |  |  |  |  |  |
| 4 | 凡事我都会积极争取。 |  |  |  |  |  |
| 5 | 无论碰到多大的困难，我都会勇往直前。 |  |  |  |  |  |
| 6 | 我觉得人生充满价值。 |  |  |  |  |  |
| 7 | 无论多辛苦，我都会坚持下去。 |  |  |  |  |  |
| 8 | 我能感受到身边的人给我带来的支持。 |  |  |  |  |  |
| 9 | 每件事，我都从好的方面看。 |  |  |  |  |  |
| 10 | 在追寻目标的时候，我会坚持下去。 |  |  |  |  |  |
| 11 | 我相信付出一定会有收获。 |  |  |  |  |  |
| 12 | 只要可以达成目标，我愿意花多一点时间。 |  |  |  |  |  |
| 13 | 我能以积极的态度面对难关。 |  |  |  |  |  |
| 14 | 我会不停学习新事物。 |  |  |  |  |  |
| 15 | 我坚持自己的信念。 |  |  |  |  |  |
| 16 | 我会视每次失败为学习机会。 |  |  |  |  |  |

5.以下是一份关于污名化的调查问卷，请根据下列的每句话，选出最符合您真实情况的答案。*-- 新冠肺炎简易污名化量表（Short version of covid-19 stigma scale）*

| 序号 | 条目 | 强烈反对 | 反对 | 赞成 | 十分赞成 |
| --- | --- | --- | --- | --- | --- |
| 1 | 有些人一旦知道我感染过新冠肺炎，就避免接触我。 |  |  |  |  |
| 2 | 我关心的人一旦知道我感染过新冠肺炎，就不再联系我。 |  |  |  |  |
| 3 | 因为告诉朋友我感染过新冠肺炎，我失去了一些朋友。 |  |  |  |  |
| 4 | 告诉某人我感染过新冠肺炎是有风险的。 |  |  |  |  |
| 5 | 我努力保守我感染过新冠肺炎的这个秘密。 |  |  |  |  |
| 6 | 我非常谨慎告诉谁我感染过新冠肺炎。 |  |  |  |  |
| 7 | 感染过新冠肺炎的人被人嫌弃。 |  |  |  |  |
| 8 | 大多数人认为感染过新冠肺炎的人很脏。 |  |  |  |  |
| 9 | 大多数人对感染过新冠肺炎的人感到不舒服。 |  |  |  |  |
| 10 | 我感到内疚，因为我感染过新冠肺炎。 |  |  |  |  |
| 11 | 人们对新冠肺炎的态度使我对自己感到更糟。 |  |  |  |  |
| 12 | 我觉得自己不如别人好，因为我感染过新冠肺炎。 |  |  |  |  |
